# Breaking the Afrotropical boundary: Discovery of *Planochelas* in Asia reveals transcontinental distribution, with a new bark-dwelling species from China (Araneae, Trachelidae)

**DOI:** 10.3897/BDJ.14.e182804

**Published:** 2026-03-03

**Authors:** Yujin Wang, Yingying Shi, Congzheng Li, Chi Jin, Liangkai Luo, Keke Liu

**Affiliations:** 1 Developmental Research Institute of Modern Agriculture, Jinggangshan University, Ji'an, China Developmental Research Institute of Modern Agriculture, Jinggangshan University Ji'an China; 2 School of Landscape and Ecological Engineering, Hebei University of Engineering, Handan, China School of Landscape and Ecological Engineering, Hebei University of Engineering Handan China; 3 Ji’an No.1 High School, Ji'an, China Ji’an No.1 High School Ji'an China

**Keywords:** Afrotropical Region, dark sac-spiders, Jiangxi Province, taxonomy

## Abstract

**Background:**

Arboreal microhabitats, such as tree bark, remain poorly studied in subtropical China. During exploratory surveys in Jiangxi, specimens of the trachelid spider genus *Planochelas* — previously known only from Africa — were collected, representing its first record in Asia. This finding disproves the genus’ assumed endemic status and underscores the value of surveying overlooked microhabitats for biogeographic discovery.

**New information:**

The spider genus *Planochelas* Lyle & Haddad, 2009 is reported for the first time outside the Afrotropical Region, based on material collected in Ji’an City, Jiangxi Province, south-eastern China. A new species, *Planochelas jingda* Liu, **sp. nov.**, is described and illustrated. Specimens were collected from beneath the bark of *Platanus* spp. (e.g. *P. acerifolia*, *P. occidentalis* and *P. orientalis*), confirming the arboreal and cryptic habits of the genus. This discovery significantly expands the known distribution of *Planochelas* and challenges its previous status as a strictly Afrotropical endemic. The finding underscores the importance of targeted microhabitat sampling for revealing overlooked spider diversity and contributes to the understanding of the biogeography of the family Trachelidae in the Oriental Region.

## Introduction

The spider family Trachelidae (Araneae) currently comprises 315 valid species from 29 genera worldwide (World Spider Catalog 2025). Historically treated as a subfamily within Corinnidae, it was elevated to family rank by Ramírez (2014) based on phylogenetic evidence. Trachelid spiders are morphologically diverse, particularly in leg spination and genitalic morphology, suggesting considerable ecological or evolutionary radiation within the group. The family exhibits a broad, but heterogeneous global distribution: some genera, such as *Orthobula* Simon, 1897 and *Trachelas* L. Koch, 1872 are widespread across the Palaearctic, Oriental and Neotropical Regions, whereas others, like *Afroceto* Lyle & Haddad, 2010 and *Fuchiba* Haddad & Lyle, 2008 are endemic to the Afrotropical Region, while *Utivarachna* Kishida, 1940 is largely restricted to Southeast Asia (World Spider Catalog 2025). This complex biogeographic pattern makes Trachelidae an interesting group for investigating historical dispersal and regional diversification in spiders.

The genus *Planochelas* Lyle & Haddad, 2009 is a distinctive trachelid lineage, characterised by a dorsoventrally flattened carapace and abdomen, the general absence of leg spines and ventral cusps (except in *P. purpureus* Lyle & Haddad, 2009) and a uniquely shaped male femoral apophysis on the palp (Lyle and Haddad 2009, Khoza and Lyle 2019). The genus was originally described from three new species collected by canopy fogging in West and Central African tropical forests (Lyle and Haddad 2009). Subsequently, Khoza and Lyle (2019) found four new species from the Democratic Republic of the Congo and South Africa, bringing the total to seven species and extending the known range of the genus to southern Africa. All described species are presumably arboreal (except the ground-dwelling *P. purpureus*) and were predominantly collected via canopy fogging, highlighting their association with forest canopies (Khoza and Lyle 2019). Until now, *Planochelas* has been considered strictly endemic to the Afrotropical Region.

In the present study, we report the first record of *Planochelas* outside Africa. Specimens were collected under the bark of large, mature *Platanus* spp. trees on the campus of Jinggangshan University in Ji’an City, Jiangxi Province, south-eastern China. This new species is described here as *Planochelas jingda* **sp. nov.**. The discovery of this bark-dwelling species confirms the arboreal and cryptic habits of the genus and extends its known distribution from the Afrotropical to the Oriental Region (Khoza and Lyle 2019), thus rejecting the previous notion of its strict Afrotropical endemism. This finding not only increases the taxonomic diversity of Trachelidae in Asia, but also brings important biogeographic questions regarding the origin and historical dispersal of the genus. Furthermore, it underscores the value of targeted sampling in microhabitats, such as under tree bark, for uncovering overlooked spider diversity in Asian subtropical forests.

## Materials and methods

Specimens were examined using a Jiangnan SZ6100 stereomicroscope. Male and female copulatory organs were dissected and examined in 80–85% ethanol. The epigyne was cleaned with pancreatin (Álvarez-Padilla and Hormiga 2007). All specimens were photographed with a KUY NICE CCD camera, equipped with an Olympus CX43 compound microscope. For SEM imagings, the specimens were dried under natural conditions, coated with gold with a ETD-2000 instrument and then photographed with a Zeiss EVO LS15 scanning electron microscope.

All measurements were made using a stereomicroscope (AxioVision SE64 rel. 4.8.3) and are given in millimetres. Leg measurements are given as total length (femur, patella, tibia, metatarsus and tarsus). All examined specimen materials are deposited in the Animal Specimen Museum, College of Life Science, Jinggangshan University, Ji’an, China (ASM-JGSU). Terminology of the male palp and female genitalia follow Lyle and Haddad (2009) and Khoza and Lyle (2019).

The abbreviations used in the figures are as follows: ALE − anterior lateral eye, AME − anterior median eye, CO − copulatory openings, Con − conductor, CT – connecting tube, DFA − dorsal femur apophysis, Em − embolus, FD − fertilisation duct, MOA − median ocular area, MS – median septum, PLE − posterior lateral eye, PME − posterior median eye, RFA – retrolateral femoral apophysis, SD − sperm duct, ST1 − spermatheca 1, ST2 − spermatheca 2.

## Taxon treatments

### Planochelas jingda

Liu sp. nov.

C2789D48-CD8D-51AD-ABA8-D6C2AFDA3068

A591D768-B0ED-4672-8301-3F4B8B033525

#### Materials

- **Type status:** Holotype. **Occurrence:** catalogNumber: ASM-JGSU-Ara6637; recordedBy: Wang Yu-jin; individualCount: 1; sex: male; lifeStage: adult; occurrenceID: C0C0C398-229E-57A9-A42F-26AAFE953199; **Taxon:** scientificName: *Planochelas jingda* sp. nov.; **Location:** country: China; stateProvince: Jiangxi; locality: Ji'an City, Qingyuan District, Jinggangshan University; verbatimElevation: 87 m; verbatimCoordinates: 27°06'48.20"N, 115°01'29.01"E; georeferenceProtocol: GPS; **Event:** samplingProtocol: handing; eventDate: 08/11/2025
- **Type status:** Paratype. **Occurrence:** catalogNumber: ASM-JGSU-Ara6606; recordedBy: Wang Yu-jin; individualCount: 1; sex: female; lifeStage: adult; occurrenceID: C24A4931-BA70-5804-B4E9-064FBEF2135C; **Taxon:** scientificName: *Planochelas jingda* sp. nov.; **Location:** country: China; stateProvince: Jiangxi; locality: Ji'an City, Qingyuan District, Jinggangshan University; verbatimElevation: 87 m; verbatimCoordinates: 27°06'48.20"N, 115°01'29.01"E; georeferenceProtocol: GPS; **Event:** samplingProtocol: handing; eventDate: 02/11/2025
- **Type status:** Paratype. **Occurrence:** catalogNumber: ASM-JGSU-Ara6638; recordedBy: Wang Yu-jin; individualCount: 1; sex: female; lifeStage: adult; occurrenceID: F64F8B35-6216-546B-B19A-A9AF071CF8EF; **Taxon:** scientificName: *Planochelas jingda* sp. nov.; **Location:** country: China; stateProvince: Jiangxi; locality: Ji'an City, Qingyuan District, Jinggangshan University; verbatimElevation: 87 m; verbatimCoordinates: 27°06'48.20"N, 115°01'29.01"E; georeferenceProtocol: GPS; **Event:** samplingProtocol: handing; eventDate: 08/11/2025
- **Type status:** Paratype. **Occurrence:** catalogNumber: ASM-JGSU-Ara7067; recordedBy: Wang Yu-jin; individualCount: 1; sex: male; lifeStage: adult; occurrenceID: 72C5E467-9767-548A-A41B-98F369E1A07D; **Taxon:** scientificName: *Planochelas jingda* sp. nov.; **Location:** country: China; stateProvince: Jiangxi; locality: Ji'an City, Qingyuan District, Jinggangshan University; verbatimElevation: 73 m; verbatimCoordinates: 27°06'43.78"N, 115°01'17.95"E; georeferenceProtocol: GPS; **Event:** samplingProtocol: handing; eventDate: 30/11/2025
- **Type status:** Paratype. **Occurrence:** catalogNumber: ASM-JGSU-Ara7147; recordedBy: Wang Yu-jin; individualCount: 1; sex: female; lifeStage: adult; occurrenceID: B17F1130-439B-5434-A87E-598D32A73F16; **Taxon:** scientificName: *Planochelas jingda* sp. nov.; **Location:** country: China; stateProvince: Jiangxi; locality: Ji'an City, Qingyuan District, Jinggangshan University; verbatimElevation: 87 m; verbatimCoordinates: 27°06'48.20"N, 115°01'29.01"E; georeferenceProtocol: GPS; **Event:** samplingProtocol: handing; eventDate: 5/11/2025
- **Type status:** Paratype. **Occurrence:** catalogNumber: ASM-JGSU-Ara7148; recordedBy: Wang Yu-jin, Zhang Bin; individualCount: 1; sex: female; lifeStage: adult; occurrenceID: 4934E59E-11C7-5640-88F5-49D29E54366B; **Taxon:** scientificName: *Planochelas jingda* sp. nov.; **Location:** country: China; stateProvince: Jiangxi; locality: Ji'an City, Qingyuan District, Jinggangshan University; verbatimElevation: 71 m; verbatimCoordinates: 27°06′49.87″N, 115°01′48.74″E; georeferenceProtocol: GPS; **Event:** samplingProtocol: handing; eventDate: 15/11/2025
- **Type status:** Paratype. **Occurrence:** catalogNumber: ASM-JGSU-Ara7149; recordedBy: Wang Yu-jin; individualCount: 1; sex: male; lifeStage: adult; occurrenceID: C451F294-55D2-54A7-A148-C83A2062F4E9; **Taxon:** scientificName: *Planochelas jingda* sp. nov.; **Location:** country: China; stateProvince: Jiangxi; locality: Ji'an City, Qingyuan District, Jinggangshan University; verbatimElevation: 73 m; verbatimCoordinates: 27°06'43.78"N, 115°01'17.95"E; georeferenceProtocol: GPS; **Event:** samplingProtocol: handing; eventDate: 30/11/2025

#### Description

**Figure 1.**
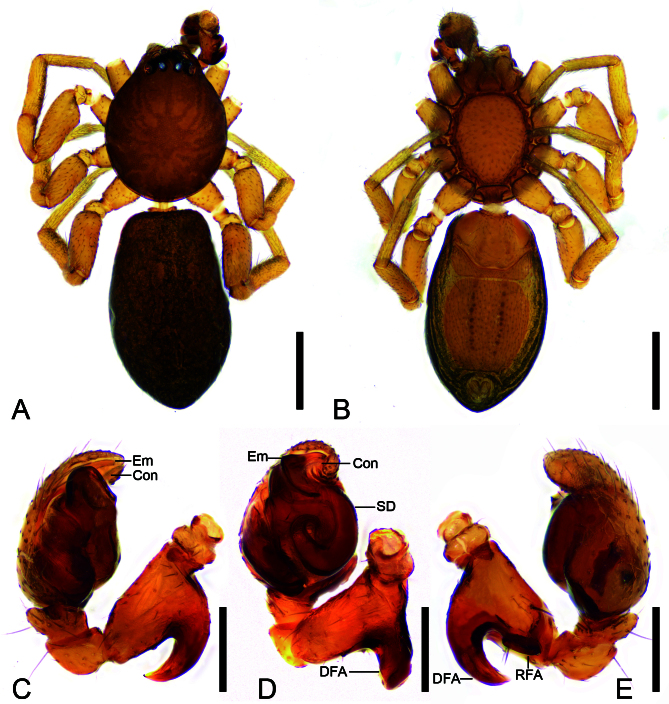
*Planochelas jingda* sp. nov., male holotype. **A** habitus, dorsal view; **B** same, ventral view; **C** palp, prolatero-ventral view; **D** same, ventral view; **E** same, retrolateral view. Scale bars: 0.5 mm (A, B); 0.1 mm (C–E).

**Male** (holotype) (Figs 1, 4A−C). Total length 2.43. Carapace (Fig. 1A and B) length 1.01, width 0.83, flattened, with radial lines of fine pits on surface. Clypeus height 0.04. Eye (Fig. 1A and B) diameters and interdistances: AME 0.07, ALE 0.06, PME 0.07, PLE 0.07; AME–AME 0.02, ALE−AME 0.01, PME–PME 0.06, PME−PLE 0.03, AME−PME 0.03, AME−PLE 0.07, ALE−ALE 0.14, PLE−PLE 0.22, ALE−PLE 0.03. MOA 0.17 long, front width 0.12, back width 0.19. Chelicerae, with sharp fangs, three promarginal and two retromarginal teeth. Endites approximately twice as long as wide, anteriorly with dense maxillar hair tuft, medially with distinct constriction. Labium trapezoidal, as long as wide, anteriorly with five to six strong setae. Sternum length 0.51, width 0.40, oval, longer than wide, anteromedially rounded, laterally with subtriangular extentions, posterior end truncated. Leg (Fig. 1A and B) measurements: I 2.15 (0.75, 0.32, 0.48, 0.34, 0.26); II 2.29 (0.79, 0.32, 0.51, 0.39, 0.28); III 1.78 (0.62, 0.21, 0.35, 0.38, 0.22); IV 2.36 (0.82, 0.31, 0.47, 0.41, 0.35); formula: 4213. Leg spination: I Ti: d2; II Ti: d2; III Fe: d1; Ti: v1; IV Fe: d2; Pa: d1; Ti: v1. Abdomen (Fig. 1A and B) 1.42 long, 0.81 wide, scutum covering entire dorsum, surface smooth, with scattered short, fine setae; venter with three sclerites, including trapezoidal epigastric sclerite, boat-shaped medial sclerite and large ovoid posterior sclerite. Spinnerets very short, with many short thick setae around surface.

Colouration (Fig. 1). Carapace, chelicerae, endites, sternum and labium reddish-brown. Legs yellow to dark brown. Abdomen dark-brown, dorsally mottled, venter with eleven to twelve pairs of brown dots medially.

**Figure 2.**
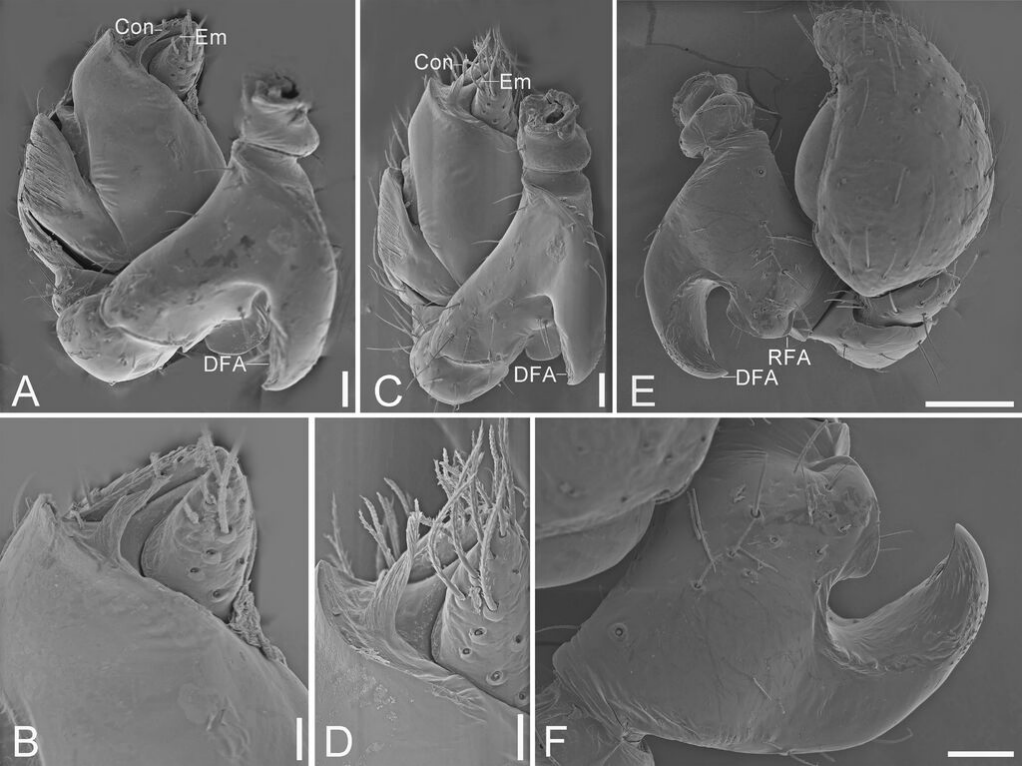
SEMs of *Planochelas jingda* sp. nov., male palp, paratype. **A** palp, prolatero-ventral view; **B** same, detail of Con and Em; **C** same, ventral view; **D** same, detail of Con and Em; **E** same, retrolateral view; **F** same, detail of DFA and RFA. Scale bars: 40 um (A, C, F); 20 um (B, D); 100 um (E).

Palp (Fig. 1C−E and Fig. 2). Dorsal femoral apophysis thick hook-shaped, very strong, with sharp apex, as long as femur. Retrolateral femoral apophysis short and blunt. Patella and tibia without apophysis, former partly encircling latter. Sperm duct S−shaped in ventral view. Embolus short, arising at end of tegulum, located at the apical cymbial groove, with broad base and slightly curved apex. Conductor membranous, as long as 2/3 embolic length, with a clavate base.

**Figure 3.**
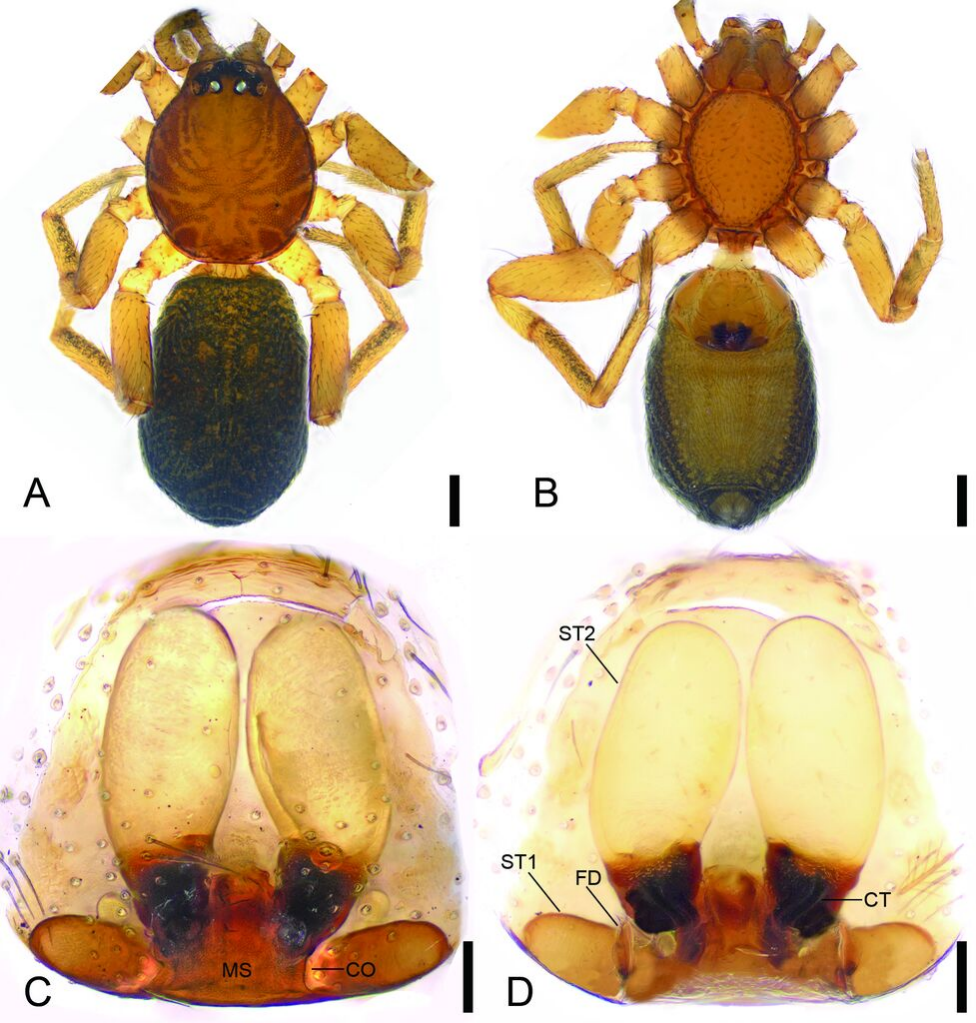
*Planochelas jingda* sp. nov., female paratype. **A** habitus, dorsal view; **B** same, ventral view; **C** epigyne, ventral view; **D** epigyne, dorsal view. Scale bars: 0.2 mm (A, B), 0.05 mm (C, D).

**Figure 4.**
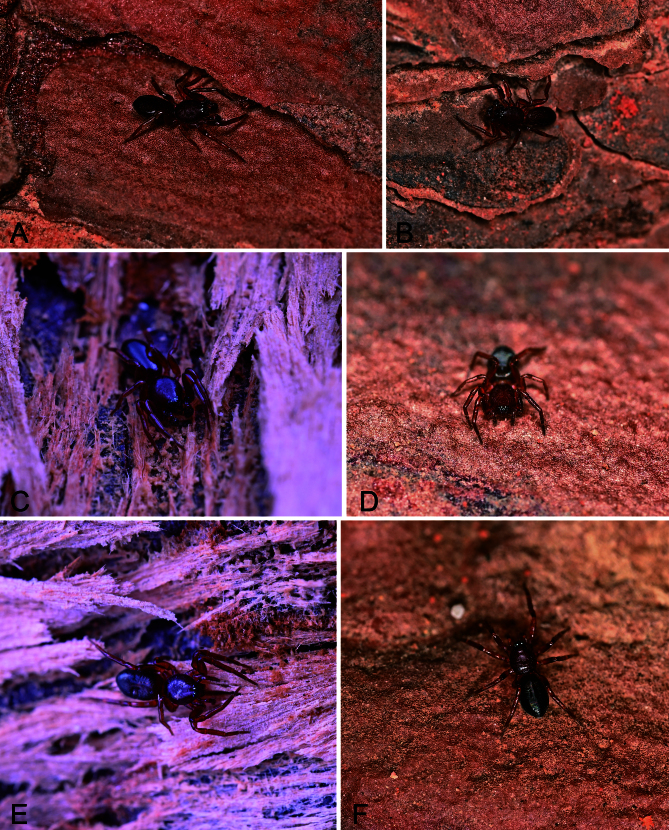
*Planochelas jingda* sp. nov., photos of living specimen **A–C** male; **D–F** female.

**Female** (Paratype) (Fig. 3 and Fig. 4D−F). As in male, except as noted. Total length 2.46. Carapace (Fig. 3A and B) length 1.00, width 0.82. Sternum length 0.68, width 0.51. Clypeus height 0.03. Eye (Fig. 3A and B) diameters and interdistances: AME 0.06, ALE 0.08, PME 0.05, PLE 0.08; AME–AME 0.02, ALE−AME 0.02, PME–PME 0.08, PME−PLE 0.03, AME−PME 0.04, AME−PLE 0.11, ALE−ALE 0.14, PLE−PLE 0.25, ALE−PLE 0.02. MOA 0.15 long, front width 0.12, back width 0.20. Legs (Fig. 3A and B) measurements: I 2.17 (0.72, 0.35, 0.51, 0.34, 0.25); II 2.22 (0.73, 0.27, 0.49, 0.43, 0.3); III 1.93 (0.69, 0.21, 0.41, 0.37, 0.28); IV 2.81 (0.90, 0.42, 0.58, 0.56, 0.35). Leg spination: I Pa: d2; Ti: d3, pl1; II Pa: d1; Ti: d3, pl1; III Fe: d1; Ti: v1; IV Fe: d2; Pa: d1; Ti: v1. Abdomen (Fig. 3A and B) 1.46 long, 0.98 wide, without scutum and sclerite.

Colouration (Fig. 3A and B). Abdominal dorsum with four to five chevrons posteriorly.

Epigyne (Fig. 3C and D). Epigynal plate as long as wide. Moderately sclerotised in posterior medial area, with broad median septum. Copulatory openings horseshoe-shaped, situated posterolaterally. Vulvae with a pair of oval spermatheca 1, a pair of large sausage-shaped spermatheca 2 and a pair of C-shaped connecting tubes between them. Fertilisation ducts leaf-shaped, directed anteriorly.

#### Diagnosis

Males of the new species resemble those of *Planochelas botulus* Lyle & Haddad, 2009 (Lyle and Haddad (2009): 93, figs. 15−16) and *P. brevis* Khoza & Lyle, 2019 (Khoza and Lyle (2019): 150, figs. 9−10) in having an S-shaped sperm duct, the anterior tegulum with an arched ridge and the slightly curved embolus situated distally on tegulum, but can be distinguished (Fig. 1C−E and Fig. 2) by the hook-shaped dorsal femoral apophysis without denticles (vs. two small, sharp denticles on the horn-like dorsal femoral apophysis in *P. botulus*; blunt denticles on short and blunt dorsal femoral apophysis in *P. brevis*), the short and blunt retrolateral femoral apophysis (vs. needle-like in *P. botulus*; absent in *P. brevis*), the digitiform conductor (vs. absent in *P. botulus* and *P. brevis*) and the embolus located within the apical cymbial groove (vs. not situated within a distinct apical groove in *P. botulus* and *P. brevis*). The female is similar to those of *P. botulus* (Lyle and Haddad (2009): 93, figs. 11−12) and *P. brevis* (Khoza and Lyle (2019): 150, figs. 12−13) in having large sausage-shaped spermatheca 2, oval spermatheca 1 and the fertilisation duct directed anteriorly, but can be separated from them (Fig. 3C and D) by the broad median septum (vs. narrow in *P. botulus* and moderate in *P. brevis*).

#### Etymology

The name is taken from Jinggangshan University, referring to the type locality; noun in apposition. Common Chinese name: 井大平蛛.

#### Distribution

**Figure 5.**
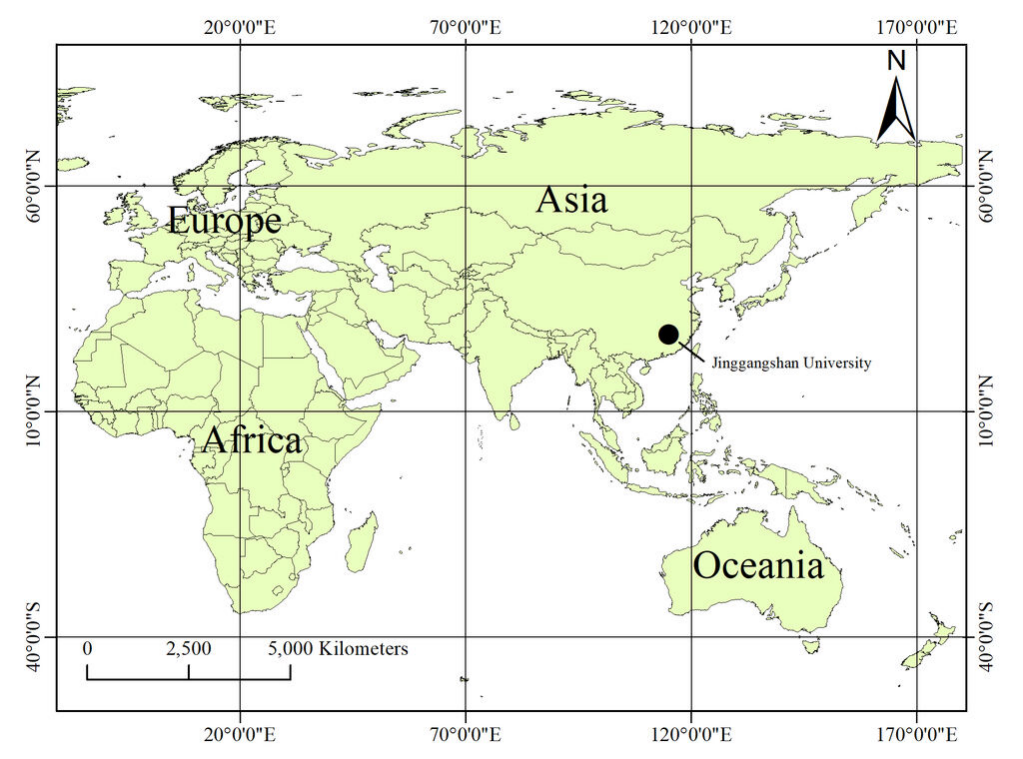
Records of *Planochelas jingda* sp. nov., from Jiangxi Province, China.

Known only from Ji'an City, in Jiangxi Province, China (Fig. 5).

#### Ecology

The specimens were collected from under the bark (Fig. 4) of plane trees (e.g. *P. acerifolia*, *P. occidentalis* and *P. orientalis*) by hand.

## Discussion

The description of a new *Planochelas* species from southern China constitutes a further addition to a growing body of taxonomic discoveries that collectively underscore the substantial, yet under-documented spider diversity within the subtropical montane ecosystems of this region. This discovery highlights the rich and heterogeneous topography of Jiangxi Province — a landscape characterised by complex hills and forested mountains that serve as crucial reservoirs of unique and endemic arachnofauna (Liu et al. 2022c, Lu et al. 2022).

It is worth mentioning that *Planochelas jingda* **sp. nov.** possesses a distinct membranous conductor, a feature not reported in any Afrotropical congeners. While this structure may have been overlooked in previous descriptions, its presence in the new species could represent a potentially significant morphological divergence that could have implications for understanding the evolution and biogeography of the genus.

The discoveries span notable taxonomic and biogeographic milestones, ranging from the first record of the genus *Ibana* in China to the discovery of *Stephanopis xiangzhouica* Liu, 2022, which represents the first record of the genus *Stephanopis* on the Asian mainland, a taxon previously known only from Australasia (Liu et al. 2022a, Liu et al. 2022b). This transcontinental disjunction not only highlights the distinctive biogeographic position of Jiangxi’s montane systems (Lyu et al. 2025), but also reveals complex historical distributional patterns and the presence of considerable cryptic diversity yet to be fully elucidated in the forests of eastern Asia.

Collectively, these accumulating discoveries — spanning new species, new generic records for China and even new continental records for genera — strongly indicate that the spider fauna of the topographically complex hills and mountains of southern China remains far from fully catalogued (Lu et al. 2022). Many lineages, particularly those associated with specialised microhabitats, such as the understorey and shrub layers of montane forests, are likely undersampled.

Future research should integrate detailed morphological study with molecular phylogenetic approaches to delimit species boundaries, elucidate the evolutionary relationships of these newly-documented taxa (including the enigmatic biogeographic occurrence of *Stephanopis* and *Planochelas* in Asia) and investigate the biogeographic histories shaping these assemblages. Such work is essential not only for establishing a stable taxonomic framework, but also for providing information for conservation priorities in these biodiverse and vulnerable montane regions.

## Supplementary Material

XML Treatment for Planochelas jingda
